# Effect of Beetroot Extract Supplementation on Serum Fatty Acid Profiles and Oxidative Stress Markers in Chronic Coronary Artery Disease Patients: A Secondary Analysis of a Randomized Controlled Trial

**DOI:** 10.1155/bmri/6654492

**Published:** 2025-12-29

**Authors:** Amirhossein Mansouri-Baseri, Mohsen Moohebati, Leila Sadat Bahrami, Hamed Tabesh, Mitra Rezaie, Mohsen Nematy, Fatemeh Davoudi, Reza Rezvani

**Affiliations:** ^1^ Department of Nutrition, Faculty of Medicine, Mashhad University of Medical Sciences, Mashhad, Iran, mums.ac.ir; ^2^ Student Research Committee, Mashhad University of Medical Sciences, Mashhad, Iran, mums.ac.ir; ^3^ Cardiovascular Research Center, School of Medicine, Mashhad University of Medical Sciences, Mashhad, Iran, mums.ac.ir; ^4^ Department of Medical Informatics, Faculty of Medicine, Mashhad University of Medical Sciences, Mashhad, Iran, mums.ac.ir; ^5^ Clinical Research Development Unit, Imam Reza Hospital, Faculty of Medicine, Mashad University of Medical Sciences, Mashad, Iran

**Keywords:** beetroot, betalains, coronary artery disease, fatty acids, nitrate, oxidative stress, vitamin C

## Abstract

**Background:**

Considering the role of a healthy diet in preventing atherogenesis, the use of beetroot as a functional food with anti‐inflammatory, antioxidant, and anti‐dyslipidemic effects due to its high nitrate content and bioactive compounds is one of the interesting approaches in coronary artery disease (CAD), a common worldwide chronic disease.

**Methods:**

This is a secondary analysis of a randomized, double‐blind, placebo‐controlled clinical trial that was conducted on 90 male (67.8%) and female (32.2%) chronic patients with CAD for 4 weeks. This secondary analysis investigated the effect of beetroot capsules within three groups on fatty acid profile, total antioxidant capacity, total oxidant status, malondialdehyde, and myeloperoxidase.

**Results:**

In subjects with 4 weeks of beetroot consumption, significant changes in the level of SFAs/PUFAs (−130 *μ*g/mL, *p* = 0.04), PA/OA (−250 *μ*g/mL, *p* = 0.02), and MPO (−9.60 U/L, *p* < 0.01) were established. In beetroot‐vitamin C group, docosahexaenoic acid (9.3 *μ*g/mL, *p* < 0.01), omega 3 (31 *μ*g/mL, *p* < 0.001), EPA + DHA (10 *μ*g/mL, *p* < 0.01), SFAs/PUFAs (−370 *μ*g/mL, *p* < 0.001), PA/OA (−360 *μ*g/mL, *p* < 0.001), TOS (−1.42 *μ*M, *p* < 0.01), and MPO (−12.42 U/L, *p* < 0.001) had notable changes.

**Conclusion:**

Beetroot capsule consumption had favorable effects on serum omega 3 fatty acids as well as TOS and MPO, the oxidant and atherogenesis factors.

## 1. Introduction

One‐third of global deaths contribute to cardiovascular diseases, particularly coronary artery disease (CAD), one of the major leading causes of noncommunicable diseases [1]. The number of premature deaths from cardiovascular disease is increasing in low‐ and middle‐income countries. CAD is the most prevalent type of organ disease because of coronary atherosclerosis, which threatens human health [1, 2]. Atherosclerosis is characterized by the formation and development of arterial plaques by arterial remodeling after subendothelial accumulation of fatty deposits, calcium, and inflammatory cells [3]. As CAD is a chronic inflammatory disease, lifestyle factors play a crucial role in the development of CAD, and healthy lifestyle interventions can be effective for the prevention and treatment of CAD [4].

Nutritional interventions can prevent atherogenesis and decrease the risk of cardiac events. They are observed as an impressive approach, mainly in patients with poor adherence to pharmacological therapies inherent in chronic diseases [5]. Nutritious foods, like green leafy vegetables and especially beetroot (*Beta vulgaris L.*), have antioxidant, anti‐inflammatory, and cardiometabolic protective effects, which contribute to high inorganic nitrate content and bioactive components such as betalains, flavonoids, carotenoids, and ascorbic acid [5, 6]. By consuming beetroot, increasing the endothelium’s nitric oxide bioavailability and the synergistic effect of polyphenols, ascorbic acid, carotenoids, and betalains can augment the conversion of nitrate to nitrite and NO [7–9]. Moreover, beetroot is red because of the presence of betalains in its composition, which have also been identified to possess high antioxidant and anti‐inflammatory properties. These compounds reduce oxidative stress, which is a common underlying cause leading to atherosclerosis and endothelial dysfunction among patients with CAD [6]. Oxidative stress is involved in an overproduction of reactive oxygen species (ROS) in excess of antioxidant defences, resulting in cellular injury and progression of CAD [10].

Vitamin C represents a powerful antioxidant that has also been under extensive research for its cardiovascular protective effects. It contributes to decelerating the process of atherosclerosis by maintaining endothelial integrity and in the relaying of signals for the synthesis of collagen, enhancing the structural integrity of arteries, trimming cholesterol and triglycerides, and preventing LDL oxidation [11]. Vitamin C also exerts an antioxidant effect through the neutralization of ROS. It reduces oxidative stress and protects against lipid peroxidation. Lipid peroxidation is one of the significant factors that lead to the formation of atherosclerotic plaques [12]. Furthermore, it has been demonstrated that vitamin C, as well as beetroot, enhance the activity and bioavailability of nitric oxide, an improving factor in vascular function [13].

Beetroot has also attracted interest in its lipid‐lowering effects the same as antioxidant and anti‐inflammatory aspects. Asgary et al. investigated the effect of beetroot juice (BRJ) on lipid profile and total antioxidant capacity (TAC). The levels of nonhigh‐density lipoprotein (HDL), low‐density lipoprotein (LDL), and total cholesterol (TC) were decreased and TAC was raised [14]. Likewise, postprandial treatment of BRJ decreased significantly TC, TG, and LDL values [15]. Moreover, freeze‐dried red beet or nitrate‐rich BRJ consumption lowered LDL cholesterol significantly in high‐risk adults for CAD [16, 17]. On the other hand, the effect of beetroot separately and in combination with vitamin C was investigated. The levels of LDL, triglycerides, and oxLDL reduced significantly, but no change was observed in HDL. So, concentrated BRJ with vitamin C promoted the reduction of blood lipids and oxidative stress in subjects with hypercholesterolemia [18].

The relationship between fatty acids, oxidative stress, and CAD has been documented in further research. First, a positive correlation between the concentration of myeloperoxidase (MPO) and the level of high sensitivity C reactive protein (hsCRP) and interleukin 6 (IL‐6) in stable patients with CAD with MPO >300 ng/mL was seen; conversely, significant hsCRP, IL‐6, and TNF‐*α* reduction in patients with MPO<300 ng/mL was observed [19]. In an animal study, BRJ pretreatment was taken further by suppressing oxidative stress, inflammation, and apoptosis in cardiac tissues, therefore improving cardiac dysfunction and structural damage [20]. Second, serum fatty acids, including SFAs and PUFAs, can also be strongly associated with lipid metabolism and inflammation. The high levels of SFA were related to a potential increase in the risk of cardiovascular disease, while PUFAs, especially omega‐3 fatty acids, have been shown to reduce inflammation and improve lipid profiles [21]. A low serum eicosapentaenoic acid (EPA) level and a low EPA/arachidonic acid (AA) ratio were associated with high vulnerability of coronary plaques [22]. While saturated fatty acids (SFAs) have proinflammatory effects via modifying the structure of the plasma membrane of cells and direct stimulation of proinflammatory signaling pathways [23].

Although extensive literature supports the cardiovascular benefits of beetroot and vitamin C supplementation, limited research has evaluated the related effects, particularly in the substantial determinants of cardiovascular events, which include serum fatty acids and oxidative stress factors. This secondary analysis aimed to investigate the individual and combined effects of beetroot and vitamin C supplementation on oxidative stress and serum levels of saturated, monounsaturated, and polyunsaturated fatty acids in patients with CAD.

## 2. Methods

This study was conducted in the Department of Nutrition, Faculty of Medicine, Mashhad University of Medical Sciences, following related guidelines and approved by the Medical Ethics Committee of Mashhad University of Medical Sciences (IR.MUMS.MEDICAL.REC.1401.157).

### 2.1. Trial Design and Participants

A detailed description of the based double blind, placebo‐controlled, randomized, parallel trial protocol has previously been published [24]. Eligible 90 patients with CAD aged between 35 and 65 years (median 52 years) who were diagnosed by a cardiologist were randomly allocated to one of the three intervention groups in equal ratios and received 500 mg of beetroot capsules (BRCs) or BRCs plus vitamin C or placebo three times/day for 4 weeks. The BRCs are made of gelatin and contained 500 mg of beetroot powder manufactured by freeze‐drying natural ingredients containing inorganic nitrate (3.2%), phenolics, ascorbic acid, carotenoids, and betalains. One‐third of the capsules were further supplemented with an additional 100 mg of vitamin C with no fillers or preservatives added. The participants were divided into three groups according to the block randomization method, comprising four participants in each block. The completion of blocks was performed based on body mass index (< 30, > 30), diabetes (yes/no), and gender (male/female). In order to blind the randomization, there were unique codes on the supplement boxes, which were also generated online. Until the end of the follow‐up period, the patients and investigators remain blinded. The placebo capsules were identical in frame, taste, and color to conceal the allocation adequately.

Prior to enrolling in the study and at the end of the 4‐week, a questionnaire of general information, the International Physical Activity Questionnaire, a 72‐hour food recall, and a quality of life and health questionnaire (Short Form Health Survey) were completed for each individual. Anthropometric measurements were collected, too. A comprehensive list of the foods containing a high amount of nitrate was given to the subjects, who were instructed to avoid consumption of these food items.

### 2.2. Sample Size

In this study, 30 patients in each intervention group (total *n* = 90) were the same as the population of a double‐blind, placebo‐controlled, randomized, parallel trial that was conducted at the cardiac outpatient clinic and Imam Reza Hospital related to Mashhad University of Medical Sciences, Mashhad, Iran. According to the mentioned trail, sample size calculation was performed with an effect size of 0.8 and considering Type 1 and 2 errors to be 5% and 20%, respectively (*α* = 0.05 and *β* = 0.2), with 80% power and 10% dropout. Finally, 30 participants were considered for each group. Moreover, based on the conducted test and the results obtained from the TOS factor, the effect size was calculated, which ultimately yielded a power of 90% for the question test. Other results with various tests also ultimately had a power above 80%.

### 2.3. Blood Collection and Oxidative Stress Analysis

The present study reports a secondary analysis of the serum’s individuals. Ten milliliters of venous blood sample was taken to assess the serum level of some parameters in the fasting state. The serum was separated from blood samples by 3000 g centrifugation at 25°C and kept at −80°C until analysis. Serum levels of TAC and TOS were measured using the ZellBio kit (Cat No. ZB‐TAC‐48A/ZB‐TAC‐96A), (Cat No. ZB‐TOS‐48A/ZB‐TOS‐96A) by colorimetric method of reduction oxidation. The serum MDA level was also measured using the ZellBio kit (Cat No. ZB‐MDA‐468A/ZB‐MDA‐96A) by colorimetric method. The basis for measuring MDA is the determination of the MDA‐TBA complex resulting from the reaction between malondialdehyde and thiobarbituric acid (TBA) at high temperatures. To investigate the atherogenesis marker factor MPO, the activity of this enzyme based on the reaction of tetramethylbenzidine (TMB) and hydrogen peroxide was evaluated using the Nampox kit through a colorimetric method.

### 2.4. Fatty Acids Analysis

To prepare fatty acid methyl esters (FAMEs) from serum for analysis separated from blood samples, the direct transesterification method was performed [25]. Concisely, to 10 mL glass screw‐cap tubes closed with Teflon, 200 *μ*L plasma was added, followed by the addition of 2 mL of methanol/toluene (4:1 v/v) containing BHT as an antioxidant at 0.01% w/v. To these, while vortexing, 200 *μ*L of acetyl chloride was added by a positive displacement pipette dropwise. To sealed tubes, which were heated up and cooled down for an hour, 5 mL of 6% potassium carbonate was added and immediately vortexed. It was further centrifuged at 2000 ×*g* at 4°C for 10 min. The supernatant of the toluene phase was later recovered and reserved for gas chromatography analyses [25]. In the following, an amount of 1 *μ*L extracted samples was used for injection into the gas chromatography (GC) device. The gas chromatograph (Varian 450, City, United States) was equipped with a cyanopropyl siloxane 88 (CP‐Sill 88) coated with silicon‐based polymers (polysiloxanes), polyethylene glycols, and solid adsorbent (EU) fused silica capillary column (100 m length, 0.25 mm internal diameter × 0.2 mm film thickness). Nitrogen was used as carrier gas. Qualitative and quantitative methyl ester identification of individuals were performed by adapting to a standard FAME Mix curve containing C4–C24 (SIGMA‐ALDRICH, United States) and cross‐sectional area of each peak, respectively.

### 2.5. Statistical Analysis

The present study’s results were analyzed using SPSS software, version 26. Data analysis for normality of distribution was performed using the Shapiro–Wilk test. The baseline demographic and clinical characteristics of the three study groups were compared using the chi‐square test. Comparisons between dose groups were examined by one‐way ANOVA with the Tukey multiple comparisons test for normally distributed data and its nonparametric counterpart of the Kruskal–Wallis test.

The test applied in this study is a parametric test in the form of normally distributed data of a paired sample *t*‐test, and its nonparametric test, Wilcoxon, in comparing the variation within each group. The *p* value of < 0.05 was considered statistically significant.

## 3. Results

### 3.1. Characteristics of Participants

**Figure 1:**
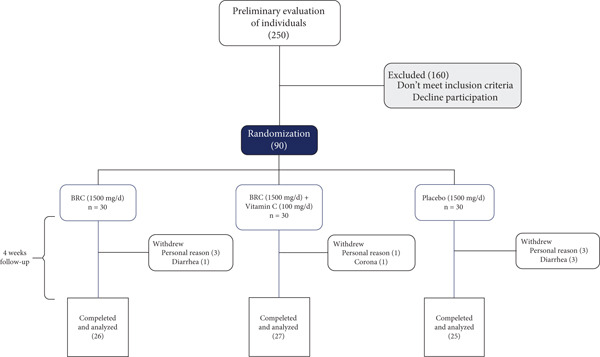
Flow diagram of study enrolment to the analysis. Abbreviation: BRC, beetroot capsule.

Ninety individuals who expressed interest in the study and were diagnosed with chronic CAD met the inclusion criteria. They were randomly assigned into three groups based on stratified blocks: Group A: red beet (*n* = 30), Group B: red beet + vitamin C (*n* = 30), and Group C: placebo (*n* = 30) in a 1:1:1 ratio. In our study, 12 patients (13%) were excluded during the study (four due to contracting COVID‐19 during the study, one due to diarrhea following capsule consumption, six due to unwillingness to continue because of the COVID‐19 peak, and one due to travel), and finally, 78 patients, including 26 in group A, 27 in group B, and 25 in group C, completed the study over a period of 4 weeks (Figure 1).

Participants’ characteristics are shown in Table 1. The overall mean age of the participants in this study is 58.24 ± 6.8, and out of the 90 patients participating in this study, 61 (67.8%) were men and 29 (32.2%) were women. Out of all the patients, 50% had a history of hypertension, 31% had diabetes mellitus, and 42% were dyslipidemic subjects. There were no significant differences in body mass index, diabetes, dyslipidemia, and smoking at baseline between the three groups. Although, there was a significant difference in hypertension between the three groups.

**Table 1:** Baseline characteristics of the participants.

		**Mean (SD)**		**p value**
	**Beetroot N = 30**	**Beetroot + VitC N = 30**	**Placebo N = 30**	
Age, year	56.07 (8.7)	57.20 (9.6)	52.4 (6.9)	0.10
Sex, no. (%)				0.81
Male	9 (30)	9 (30)	11 (36.7)	
Female	21 (70)	21 (70)	19 (63.3)	
Weight, kg	75.85 (13.8)	72.66 (13)	76.98 (15)	0.48
Height, cm	167.63 (7.9)	166.5 (8.4)	167.53 (9)	0.85
Body mass index, kg/m^2^	26.97 (4.1)	26.21 (3.9)	27.37 (5.3)	0.60
Diabetes, no. (%)	10 (33.3)	9 (30)	9 (30)	0.94
Hypertension, no. (%)	21 (70)	14 (46.7)	10 (33.3)	0.02
Dyslipidemia, no. (%)	16 (53.3)	10 (33.3)	12 (42.2)	0.28
Smoking, no. (%)	7 (23.3)	9 (30)	5 (16.7)	0.47

*Note:* The continuous variables were presented as mean and standard deviation, whereas the categorical variables were presented as percentages. Chi‐square tests for categorical variables and one‐way ANOVAs for normally distributed variables were conducted to find the differences in the baseline data.

### 3.2. Medications and Anthropometry

As stated in based RCT, among the patients participating in this study, a total of 87.8% received aspirin, and 81.1% received statins, which were part of the treatment protocol for CAD. Additionally, 69.7% reported using antihypertensive medications, and 35.6% reported using diabetes management medications. It was demonstrated that there were no significant differences in these medications between interventions, except for the use of antihypertensive medications.

Likewise, there were no significant differences in anthropometric factors for instance weight, waist circumference, hip circumference, neck circumference, fat mass, lean mass, and ABSI (a body shape index) at baseline and after 4 weeks of supplementation between groups.

### 3.3. Dietary Intake and Physical Activity

The study found no significant differences in the 72‐hour dietary intake (calories, carbohydrates, protein, total fat, monounsaturated fatty acids, polyunsaturated fatty acids, SFAs, soluble fiber, insoluble fiber, dietary fiber, sodium, potassium, magnesium and vitamin C) and MET (metabolic equivalent of task) score between the three groups before and after the interventions (sup).

### 3.4. SFAs

After 4 weeks of interventions, among the SFAs between the three groups, only the differences in the means of lauric acid (C12:0); *p* < 0.001 and pentadecanoic acid (C15:0); *p* = 0.02 were significant. Relative to baseline, beetroot interventions increased very long‐chain saturated fatty acids (VLCFAs). BRC and BRC + Vit C augmented behenic acid (C22:0); *p* = 0.01 and both behenic acid (C22:0); *p* = 0.01 and lignoceric acid (C24:0); *p* = 0.01, respectively. No significant differences were observed in other and the total SFAs between the three groups after the study (Table 2).

**Table 2:** Effects on saturated fatty acids.

	**Participant group, mean change from baseline (95% CI)** ^a^, ***μ*g/mL**			**Between-group comparison** ^b^			
	**Beetroot N = 26**	**Beetroot + VitC N = 27**	**Placebo N = 25**	**BRC vs. BRC + Vit C**	**BRC vs. placebo**	**BRC + Vit C vs. placebo**	**p value**
Medium and long chain SFAs							
Lauric acid (C12:0)	3.5 (−0.1 to 7.1)	−4.4 (−6.1 to −2.7)∗∗∗	−1.2 (−5.5 to 3.0)	**< 0.001**	**0.001**	0.90	***< 0.001***
Myristic acid (C14:0)	−4.9 (−10.6 to 0.7)	2.2 (−4.3 to 8.8)	−0.4 (−7.1 to 6.3)	0.08	0.25	0.87	0.08
Pentadecanoic acid (C15:0)	1.3 (−0.5 to 3.1)	1.3 (−1.0 to 3.7)	0.3 (−1.7 to 2.4)	*0.03*	0.97	0.06	*0.02*
Palmitic acid (C16:0)	−129.6 (−293.3 to 34.1)	−75.6 (−212.8 to 61.5)	−125.3 (−265.2 to 14.5)	0.35	0.77	0.78	0.39
Stearic acid (C18:0)	−65.3 (−143.0 to 12.3)	−36.9 (−100.5 to 26.6)	−75.9 (−138.2 to −13.8)∗	0.86	0.99	0.88	0.84
Arachidic acid (C20:0)	0.5 (−1.0 to 2.0)	−3.9 (−11.4 to 3.4)	−3.8 (−11.9 to 4.2)	0.96	0.94	0.99	0.94
VLCFAs							
Behenic acid (C22:0)	5 (0.6 to 9.5)∗	8.1 (2.2 to 14.0)∗	−0.3 (−9.1 to 8.4)	0.91	0.25	0.11	0.11
Lignoceric acid (C24:0)	1.3 (−4.4 to 7.0	9.5 (2.0 to 17.2)∗	1.8 (−7.6 to 11.4)	*0.04*	0.36	0.58	0.05
VLCFAs	6.8 (−3.3 to 17.1)	13.6 (−0.5 to 27.9)	−2.2 (−23.0 to 18.5)	0.25	0.99	0.28	0.20
SFAs	−215.9 (−472.1 to 40.2)	−120.5 (−328.0 to 86.9)	−218.6 (−439.0 to 1.7)	0.43	0.87	0.75	0.46

Abbreviations: BRC, beetroot capsule; SFAs, saturated fatty acids; VLCFAs, very long‐chain saturated fatty acids.

^a^Changes in outcome variables within groups were assessed using paired *t*‐tests for variables with a normal distribution. ∗*p* < 0.05, ∗∗*p* < 0.01, ∗∗∗*p* < 0.001: notable differences from baseline.

^b^For comparisons between groups, one‐way ANOVAs were utilized, followed by post hoc Tukey tests for normally distributed variables. *p* < 0.05 and *p* < 0.01 are shown in italics and bold.

### 3.5. Unsaturated Fatty Acids

Among the investigated monounsaturated fatty acids, only a significant difference in the oleic acid (C18:1n9c); *p* = 0.01, between three groups was noticed after supplementation. There were no significant differences in other MUFAs between and within groups.

Based on the obtained results, between intervention groups and after the follow‐up, the differences of two polyunsaturated fatty acids (PUFAs), including linoleic acid (C18:2n6c); *p* = 0.02, docosahexaenoic acid (C22:6n3); *p* < 0.01, and the sum of PUFAs; *p* = 0.04, and omega‐3 fatty acids; *p* < 0.01 were significant. As compared with baseline, docosahexaenoic acid (C22:6n3) and omega 3 fatty acids were increased in BRC + Vit C (9.3, *p* = 0.006 and 31, *p* < 0.001) group after 4 weeks. Moreover, this group let to a considerable augmentation in linoleic acid (C18:2n6c); *p* = 0.053. The differences of other unsaturated fatty acids between three groups were not significant after interventions (Table 3).

**Table 3:** Effects on unsaturated fatty acids.

	**Participant group, mean change from baseline (95% CI)** ^a^, ***μ*g/mL**			**Between-group comparison** ^b^			
	**Beetroot N = 26**	**Beetroot + VitC N = 27**	**Placebo N = 25**	**BRC vs. BRC + Vit C**	**BRC vs. placebo**	**BRC + Vit C vs. placebo**	***p* value**
MUFAs							
Palmitoleic acid (C16:1)	−9.9 (−20.5 to 0.5)	−2.6 (−13.1 to 7.7)	−7.3 (−18.9 to 4.2)	0.26	0.43	0.96	0.26
Oleic acid (C18:1n9c)	−41 (−145.5 to 63.3)	99.5 (−26.6 to 225)	−41.4 (−153 to 71)	*0.02*	0.99	*0.03*	*0.01*
Nervonic acid (C24:1)	−1.1 (−4.1 to 1.8)	1.9 (−1.1 to 4.9)	0.5 (−4.8 to 5.9)	0.28	0.49	0.93	0.28
Total MUFAs	−51.8 (−169.0 to 65.2)	99.1 (−38.9 to 237.2)	−47.3 (−167.7 to 73.1)	0.07	0.97	0.13	0.05
PUFAs							
*ω*‐6							
Linoleic acid (C18:2n6c)	−87.7 (−253.0 to 77.5)	177 (−2.3 to 357.2)	−83.6 (−203.7 to 36.4)	*0.02*	0.74	0.12	*0.02*
*γ*‐Linolenic acid (C18:3n6)	−0.6 (−4.7 to 3.4)	1.1 (−2.0 to 4.4)	<0.1 (−6.7 to 6.7)	0.93	0.63	0.83	0.65
Dihomo‐*γ*‐linolenic acid (C20:3n6)	−6.6 (−19.0 to 5.8)	3.8 (−6.9 to 14.5)	−8.3 (−18.0 to 1.2)	0.65	0.99	0.66	0.60
Arachidonic acid (C20:4n6)	−54 (−106.2 to −1.9)	−28.2 (−65.5 to 9.0)	−67.6 (−121.2 to −14.0)∗	0.86	0.84	0.99	0.82
Total omega 6	−141.8 (−366.1 to 82.3)	152.1 (−57.5 to 361.7)	−160.2 (−332.0 to 11.6)	0.04	0.77	0.22	0.05
*ω*‐3							
*α*‐Linolenic acid (C18:3n3)	3.5 (−9.6 to 16.8)	4.2 (−0.6 to 9.2)	−2.7 (−6.7 to 1.2)	0.99	0.67	0.68	0.63
Eicosapentaenoic acid (C20:5n3)	1.3 (−0.3 to 3.0)	1.1 (−0.5 to 2.8)	1.7 (−0.1 to 3.6)	0.93	0.63	0.82	0.65
Docosahexaenoic acid (C22:6n3)	−2.8 (−7.9 to 2.2)	9.3 (2.9 to 15.8)∗∗	−0.5 (−6.6 to 5.6)	*0.01*	0.98	*0.02*	**0.008**
Total omega 3	9 (−9.4 to 27.6)	31 (19.5 to 42.5)∗∗∗	0.3 (−9.8 to 10.5)	0.07	0.49	**0.004**	**0.004**
Total PUFAs	−130 (−371.2 to 111.1)	183 (−37.3 to 403.4)	−160.9 (−342.0 to 20.1)	*0.04*	0.85	0.16	*0.04*

Abbreviations: BRC, beetroot capsule; MUFAs, monounsaturated fatty acids; PUFAs, polyunsaturated fatty acids.

^a^Changes in outcome variables within groups were assessed using paired t‐tests for variables with a normal distribution. ∗*p* < 0.05, ∗∗*p* < 0.01, ∗∗∗*p* < 0.001: notable differences from baseline.

^b^For comparisons between groups, one‐way ANOVAs were utilized, followed by post hoc Tukey tests for normally distributed variables. *p* < 0.05 and *p* < 0.01 are shown in italics and bold.

### 3.6. Fatty Acid Indices

It was indicated that there were no significant differences in these indices between comparing groups, except for EPA + DHA, *p* = 0.01 after 4 weeks. After 4 weeks, BRC + Vit C led to a notable increase in EPA + DHA (10.4, *p* = 0.006), SFA/PUFA (−232.1, *p* < 0.001), and PA/OA (−359.3, *p* < 0.001) while the placebo did not cause any changes (Table 4).

**Table 4:** Effects on fatty acid indices.

	**Within-group comparison** ^a^			**Between-group comparison** ^b^
	**Beetroot N = 26**	**Beetroot + VitC N = 27**	**Placebo N = 25**	***p* value**
EPA + DHA	0.59^e^	**< 0.01** ^d^	0.70^d^	*0.01*
SFA/PUFA	*0.04* ^e^	**< 0.001** ^e^	0.19^e^	0.28
Omega 6/Omega 3	**< 0.01** ^e^	*0.01* ^e^	*0.01* ^e^	0.19
PA/OA	*0.02* ^e^	**< 0.001** ^e^	0.06^e^	0.47
EPA/AA^c^	*0.02*	0.25	*0.01*	0.74
DHA/AA^c^	0.24	*0.02*	*0.01*	0.23
Omega 3 Index^c^	0.10	*0.02*	*0.01*	0.50
Total fatty acid	0.17^e^	0.54^d^	0.07^e^	0.11

Abbreviations: AA, arachidonic acid; DHA, docosahexaenoic acid; EPA, eicosapentaenoic acid; OA, oleic acid; PA, palmitic acid; PUFAs, polyunsaturated fatty acids; SFAs, saturated fatty acids.

^a^Paired *t* tests were performed for variables with a normal distribution and paired Wilcoxon tests for abnormally distributed variables.

^b^For comparisons between groups, one‐way ANOVAs were utilized for normally distributed variables or Kruskal–Wallis H tests for abnormally distributed variables.

^c^Abnormal distributed variables.

^d^Increasing mean change.

^e^Decreasing mean change.

### 3.7. Oxidative Stress

The substantial differences in total oxidative status (TOS); *p* < 0.001 and myeloperoxidase (MPO); *p* < 0.01 were caused between the three groups by beetroot supplementation. MPO diminished in both BRC (60/9‐, *p* < 0.01) and BRC + Vit C (42/12‐, *p* < 0.001) groups at the 4‐week follow‐up as well as TOS in BRC + Vit C (42/1‐, *p* < 0.01). Also, a considerable increase (26.03, *p* = 0.07) in TAC (26.03, *p* = 0.07) was observed in the BRC + Vit C group. There were no considerable differences in changes in MDA level among the interventions (Table 5).

**Table 5:** Effects on oxidative stress.

	**Participant group, mean change from baseline^a^ (95% CI)**			**Between-group comparison^b^**			
	**Beetroot N = 26**	**Beetroot + VitC N = 27**	**Placebo** **N** = 25	**SBRC vs. BRC + Vit C**	**BRC vs. placebo**	**BRC + Vit C vs. placebo**	**p value**
TAC (*μ*M)	9.26 (−18.13 to 36.67)	26.03 (−3.13 to 55.20)	−9.91 (−29.50 to 9.67)	0.95	0.99	0.94	0.93
TOS (*μ*M)	0.35 (−0.34 to 1.05)	−1.42 (−2.26 to −0.59)∗∗	0.22 (−0.12 to 0.57)	**0.001**	0.96	**0.002**	**< 0.001**
MDA (*μ*M)	−2.59 (−6.14 to 0.96)	0.46 (−1.45 to 2.39)	−0.23 (−2.04 to 1.56)	0.87	0.18	0.38	0.19
MPO (U/L)	−9.60 (−15.59 to −3.61)∗∗	−12.42 (−16.73 to −8.11)∗∗∗	−1.67 (−7.08 to 3.73)	0.99	**0.004**	**0.002**	**0.001**

Abbreviations: BRC, beetroot capsule; MDA, malondialdehyde; MPO, myeloperoxidase; TAC, total antioxidant capacity; TOS, total oxidant status.

^a^Changes in outcome variables within groups were assessed using paired t‐tests for variables with a normal distribution. ∗*p* < 0.05, ∗∗*p* < 0.01, ∗∗∗*p* < 0.001: notable differences from baseline.

^b^For comparisons between groups, one‐way ANOVAs were utilized, followed by post hoc Tukey tests for normally distributed variables. *p* < 0.05 and *p* < 0.01 are shown in italics and bold.

## 4. Discussion

In this study, pentadecanoic acid, oleic acid, linoleic acid, docosahexaenoic acid, omega‐3, polyunsaturated fatty acids, EPA + DHA, and TOS in participants from the BRC group combined with vitamin C showed significant differences compared with the beetroot and placebo groups post‐intervention. Nonetheless, lauric acid in the BRC group differed significantly from the other two groups, and myeloperoxidase in the beetroot and beetroot with vitamin C groups differed significantly from the placebo group. To our knowledge, this is the first study to investigate the effects of BRC, Vitamin C, and their combination on fatty acid profiles, TOS, MDA, and MPO.

### 4.1. Impact of Interventions on Fatty Acids

#### 4.1.1. SFAs

Beetroot may exert lipid‐lowering effects through increased fat metabolism and inhibiting acetyl‐CoA carboxylase, an enzyme regulated by insulin that controls fatty acid synthesis and degradation. Furthermore, the nitrates and bioactive compounds in beetroot reduce oxidative stress in fatty membranes and LDL by lowering ROS [26, 27]. SFAs contribute to cell membrane structure and activate pro‐inflammatory pathways, associated with inflammation and atherosclerosis [28].

Studies have shown significant improvements in lipid profiles with beetroot and nitrate‐rich BRJ interventions [14, 17, 29]. An association between beetroot’s bioactive compounds and LDL oxidation prevention, thereby reducing inflammation and atherosclerosis, was noted earlier. These compounds, particularly betalains, appear to lower blood lipids, though the mechanism is unclear. Basar et al. reported notable reductions in TG (−10.32 mg/dL), TC (−15.45 mg/dL), and LDL (−11.54 mg/dL) after 2 weeks of daily BRJ (70 mL) with 1000 mg of vitamin C [18]. Findings suggested that nitrates may enhance lipid‐lowering effects in combination with vitamin C and other antioxidants, which reduce oxidative stress [30].

Clark et al. found significant positive correlations between several serum fatty acids and LDL, TC, and TG levels [31], suggesting that lower blood lipid levels may lead to reduced serum fatty acids. Thus, our study yielded contradictory findings, revealing significant decrease in lauric among beetroot with vitamin C group. Both the beetroot and beetroot with vitamin C groups demonstrated augmentation in behenic and lignoceric acids, while the placebo group showed a decrease in stearic acid. Other SFAs, including their total, did not notably change. Studies have associated higher circulating stearic acid with reduced cardiovascular mortality and LDL, particularly when replaced by palmitic acid [32, 33], indicating that beetroot may have prevented stearic acid reduction. Higher levels of very long‐chain fatty acids (arachidic, behenic, and lignoceric acids) have been associated with a 52% lower risk of CAD [34]. Moreover, a meta‐analysis by Lee et al. reported a 20% reduction in cardiovascular disease related to higher serum levels of these fatty acids [35], proposing that the increases observed in our intervention groups may indicate improvements in CAD.

Despite this, the overall levels of SFAs and other fatty acids in the present study did not change significantly, which was confirmed by Amirpour et al.’s meta‐analysis. LDL, HDL, TC, and TG levels also had no considerable differences between intervention and control groups after subgroup analyses based on health status, beetroot intake form, and type of intervention. This study suggested that beetroot may not be an effective supplement for regulating lipid profiles [36]. Our hypothesis regarding the effectiveness of beetroot and vitamin C on SFAs reduction was not confirmed; however, prolonged supplementation with enhanced bioactive compounds may yield positive results.

#### 4.1.2. Unsaturated Fatty Acids

The increase in free radicals leads to fatty acid peroxidation in LDL particles, resulting in the fragmentation of apolipoprotein B‐100 [37]. Nitrate and betaine, as beetroot complements, are suspected to have radical‐scavenging activities. Furthermore, unsaturated fatty acids, and especially PUFAs, might affect membrane fluidity and stability, whereas they execute as substrates for lipid regulators implicated in inflammation. The development of atherosclerosis is closely associated with the imbalance of pro‐ and anti‐inflammatory mechanisms. Fatty acids are essential in endothelial cell functions, and disruptions are associated with atherosclerosis and CAD [28].Omega‐3 fatty acids regulate T cell differentiation, generating prostaglandins and specialized pro‐resolving lipid mediators (SPMs), all of which support tissue repair and the resolution of inflammation [38].

Studies have examined the relationship between lipid profiles and unsaturated fatty acids in CAD. Noori et al. noted significantly lower levels of linoleic acid, eicosapentaenoic acid (EPA), and docosahexaenoic acid (DHA) in HDL lipoproteins among patients with coronary artery stenosis compared with those without stenosis, with CAD severity inversely associated with EPA and DHA in HDL [39]. The anti‐inflammatory effects of omega‐6 and omega‐3 fatty acids have been identified in suppressing atherogenic processes in endothelial cells. Lower EPA levels and EPA‐to‐AA ratios were associated with higher coronary plaque vulnerability in patients with CAD [22]. Despite the role of omega‐6 fatty acids in generating pro‐inflammatory prostaglandins, a meta‐analysis of 30 studies linked higher linoleic acid levels with a lower risk of cardiovascular events [40]. Sun et al. observed that there was a significant positive relation between serum palmitic‐to‐oleic acid ratio and increased CAD mortality risk [41]. It may prevent atherogenesis by reducing cellular saturated fats and decreasing VCAM‐1 and NF‐*κ*B activation [42]. Lamaytr et al. demonstrated the inverse relationship of CAD mortality with circulating linoleic acid [43].

Along with the protective effects of unsaturated fatty acids, our study revealed noteworthy results on the efficacy of beetroot and vitamin C, which may be attributed to the vitamin C effect, not BRJ per se. Considerable differences were observed in total PUFAs and omega‐3 fatty acids, as well as the oleic acid, docosahexaenoic acid, omega‐6, and the EPA + DHA between groups. DHA levels, the EPA + DHA, and total omega‐3 significantly increased only in beetroot‐plus‐vitamin C group. There was a significant decrease in the omega‐6/omega‐3 ratio across all groups. The palmitic/oleic acid and SFA/PUFA ratios exhibited notable reductions in both the beetroot‐only and beetroot‐plus‐vitamin C groups. Arachidonic acid decreased significantly in the placebo group, and the EPA/AA in the beetroot group and DHA/AA, Omega‐3 Index, and EPA + DHA/AA in the beetroot‐plus‐vitamin C group have significant increases. All these four indices also increased in the placebo group, possibly reflecting the reduction in arachidonic acid and total fatty acids in this group.

Given the decrease in the omega‐6 to omega‐3 ratio across all groups, it cannot be attributed solely to beetroot intervention, and based on current information, no conclusion can be drawn regarding the reduction of arachidonic acid in the placebo group. Yagi et al. demonstrated that lower serum DHA levels, positively correlated with flow‐mediated dilation (FMD), might act as a biomarker for impaired endothelial function [44]. Previous studies have also shown that DHA can suppress the expression of pro‐atherogenic and pro‐inflammatory proteins induced by cytokines in human endothelial cells [45, 46]. Based on our hypothesis, beetroot and vitamin C supplementation may increase DHA as a polyunsaturated fatty acid, with vitamin C potentially enhancing this effect. As noted, vitamin C can inhibit lipid peroxidation of fatty acids in LDL and HDL particles by neutralizing free radicals, which may reduce oxidation of unsaturated fatty acids. Moreover, previous studies have observed increases in HDL and decrease in LDL with vitamin C supplementation.

### 4.2. Impact of Interventions on Oxidative Stress Factors

#### 4.2.1. TAC and TOS

Based on animal and human studies, oxidative stress and the related pre‐inflammatory response are considered atherogenic and are attributed to reduced endothelial function. It is hypothesized that dietary nitrates induce antioxidant activity by inhibiting ROS production. ROS, especially superoxide anions, react with nitric oxide, forming peroxynitrite, which oxidizes LDL and increases pro‐inflammatory cytokines and adhesion molecules. The decrease in NO bioavailability and disruption of endothelial function are its results [47, 48]. Beetroot, rich in bioactive compounds like betalains, can eliminate ROS and induce antioxidant defenses. Animal studies show betalains protect against LDL oxidation through radical‐scavenging activity [49, 50].

Studies report significant enhancements in TAC and antioxidant enzyme levels after beetroot supplementation [14, 20, 51]. Two betalain metabolites (betanin and betanidin) reduced linoleate damage and lipid oxidation by H_2_O_2_. Betanin’s high antioxidant activity is linked to its ability to neutralize reactive radicals [52]. Beetroot nitrates also increase NO production, preventing radical formation and eliminating ROS and nitrogen species [53, 54].

In the current study, consistent with the mentioned studies, significant differences in TOS and mean differences in this factor were observed between the intervention groups. The beetroot with the vitamin C group has a significant reduction in TOS, while TAC increased substantially, though not significantly. Vitamin C appears to enhance the effects of beetroot, reducing TOS by influencing the nitrite–nitrate–nitric oxide cycle, reducing ROS, and increasing antioxidant activity. A longer intervention duration may lead to significant changes in TAC levels. Thus, we can confirm the efficacy of beetroot combined with vitamin C on TAC and TOS.

#### 4.2.2. Malondialdehyde and Myeloperoxidase

Nitrates, by increasing NO production, inhibit ROS, reducing LDL oxidation and atherogenic inflammation. The entrapment of lipoproteins containing apo B100 in the subendothelium is a key step in atherogenesis, leading to decreased NO availability, increased free radical production, and inflammation [55]. The increase in free radicals causes peroxidation of fatty acids in LDL particles and decreases polyunsaturated fatty acids, leading to the fragmentation of apolipoprotein B‐100 [37]. Beetroot betalains prevent LDL oxidation by binding to the polar head of fatty acids or apo B‐100 residues. In chronic CAD, elevated myeloperoxidase levels from leukocyte activation lead to oxidant production and plaque formation [56]. Dyslipidemia and dyslipoproteinemia lead to inflammation by increasing MPO levels, lowering apoAI and HDL, and impairing HDL function [19].

MDA and MPO levels were significantly reduced by daily BRJ and extract interventions in animal and human studies [20, 57, 58]. However, Siyar et al. found no significant changes in MDA levels after a 5‐week BRJ intervention [59].We found no significant difference in MDA levels was observed between or within the intervention groups, while MPO levels were significantly reduced in both the beetroot and beetroot with vitamin C groups. Nitrates can reduce leukocyte activity and adhesion, increasing NO production in atherosclerosis and thereby mitigating inflammation. NO can influence neutrophil CB11, and interestingly, the binding of MPO to the CB11 antigen enhances the activity of this white blood cell. The nitrate intervention could lead to at least a partial reduction in CB11 expression due to the direct suppression of MPO activity [60]. Based on our hypothesis, beetroot and vitamin C affected MPO levels, and more research is needed to explore the mechanisms involved.

### 4.3. Strengths and Limitations

To our knowledge, this study is the first to investigate the effects of beetroot on serum fatty acids and oxidative stress in patients with chronic CAD. BRCs were freeze‐dried to preserve bioactive compounds. Stratified block randomization ensured a balanced distribution of participants and control for confounders. Collaboration among cardiologists, pharmacists, biostatisticians, and nutritionists was a key strength. Because of the considerable detection of fatty acids, an efficient fatty acid sample preparation method for gas chromatography was also a benefit. However, because of the small number of hypertensive and non‐hypertensive individuals, adjustments for blood pressure were not performed, resulting in decreased statistical power.

Further studies are necessary to evaluate the long‐term effects of beetroot consumption in patients with chronic CAD, given the mild and transient gastrointestinal adverse effects found and the lack of any other specific adverse effects. It should be considered that the acceptable daily intake (ADI) of nitrate from beetroot and diet is 3.7 mg/kg body weight. Subsequent studies could also explore the impact of beetroot on the fatty acids of red blood cell membranes. Moreover, future research should examine the fatty acid profiles of LDL and HDL particles in CAD patients.

## 5. Conclusion

This study demonstrated that the consumption of 1500 mg daily of BRCs over a period of 4 weeks had beneficial effects on the levels of DHA and total omega 3 fatty acids in patients with chronic CAD. However, no effects were observed on SFAs. Additionally, this supplementation led to improvements in oxidative stress and atherogenesis markers, specifically TOS and MPO. The positive effects mentioned were more pronounced in the group receiving beetroot with vitamin C compared with the group receiving beetroot alone. Further long‐term studies are needed to confirm and clarify the effect of BRC and vitamin C on serum fatty acids and oxidative stress in CAD.

## Disclosure

All authors have read and agreed to the published version of the manuscript.

## Conflicts of Interest

The authors declare no conflicts of interest.

## Author Contributions

Conceptualization, Amirhossein Mansouri‐Baseri, Mohsen Nematy, Mohsen Moohebati, and Reza Rezvani; methodology, Amirhossein Mansouri‐Baseri, Mohsen Moohebati, Leila Sadat Bahrami, Hamed Tabesh, Mitra Rezaie, and Reza Rezvani; formal analysis, Amirhossein Mansouri‐Baseri, Hamed Tabesh, and Reza Rezvani; investigation, Amirhossein Mansouri‐Baseri, Hamed Tabesh, Mitra Rezaie, Mohsen Nematy, Fatemeh Davoudi, and Reza Rezvani; writing – original draft preparation, Amirhossein Mansouri‐Baseri, Mohsen Moohebati, Leila Sadat Bahrami, Hamed Tabesh, and Reza Rezvani; writing – review and editing, Amirhossein Mansouri‐Baseri, Leila Sadat Bahrami, Mitra Rezaie, Mohsen Nematy, and Reza Rezvani; supervision, Reza Rezvani, Mitra Rezaie, Mohsen Moohebati, and Hamed Tabesh; project administration, Reza Rezvani, Mitra Rezaie, Amirhossein Mansouri‐Baseri and Leila Sadat Bahrami.

## Funding

This study was supported by Mashhad University of Medical Sciences.

## Data Availability

Data are available on request from the corresponding author. The data are not publicly available due to confidentiality.
